# Wide Bandwidth Vibration Energy Harvester with Embedded Transverse Movable Mass

**DOI:** 10.3390/s21165517

**Published:** 2021-08-17

**Authors:** Nathan Jackson, Luis A. Rodriguez, Rahul Adhikari

**Affiliations:** Center for High Technology Materials and Mechanical Engineering Department, University of New Mexico, Albuquerque, NM 87106, USA; luis.a.rod.mil@gmail.com (L.A.R.); rahulvinda@unm.edu (R.A.)

**Keywords:** bandwidth, energy harvester, piezoelectric material, cantilever, MEMS

## Abstract

One of the biggest challenges associated with vibration energy harvesters is their limited bandwidth, which reduces their effectiveness when utilized for Internet of Things applications. This paper presents a novel method of increasing the bandwidth of a cantilever beam by using an embedded transverse out-of-plane movable mass, which continuously changes the resonant frequency due to mass change and non-linear dynamic impact forces. The concept was investigated through experimentation of a movable mass, in the form of a solid sphere, that was embedded within a stationary proof mass with hollow cylindrical chambers. As the cantilever oscillated, it caused the movable mass to move out-of-plane, thus effectively altering the overall effective mass of the system during operation. This concept combined high bandwidth non-linear dynamics from the movable mass with the high power linear dynamics from the stationary proof mass. This paper experimentally investigated the frequency and power effects of acceleration, the amount of movable mass, the density of the mass, and the size of the movable mass. The results demonstrated that the bandwidth can be significantly increased from 1.5 Hz to >40 Hz with a transverse movable mass, while maintaining high power output. Dense movable masses are better for high acceleration, low frequency applications, whereas lower density masses are better for low acceleration applications.

## 1. Introduction

With the increase in the demand for the Internet of Things, cyber-physical systems, and smart buildings, there continues to be a high interest in vibration energy harvesting to create self-sustaining systems through the harvesting and conversion of mechanical energy from the ambient environment into usable electrical energy. Most vibration energy harvesters consist of a cantilever beam as a mechanical system that oscillates due to an applied vibration from the environment. The different kinetic energy converting mechanisms include piezoelectrics, electromagnetics, electrostatics, or triboelectrics. The energy converting mechanisms are different for each of these, but all these methods rely on a similar mechanical oscillating system. Typically, these cantilevers are linear systems with high Q-factors or narrow bandwidths of <2 Hz (~1% of resonant frequency) operating at low frequencies of <250 Hz [1,2,3,4,5,6]. The narrow bandwidths allow them to have high power density, but it limits their use in real-life applications, as even a 1% change in resonant frequency will significantly reduce the amount of power harvested. Large changes in resonant frequency can occur due to small changes in the vibration source or even due to manufacturing non-uniformities of the cantilever. Therefore, wider bandwidth devices are necessary for practical applications.

Since bandwidth is one of the major challenges associated with vibration energy harvesting, it was extensively investigated. Previous attempts to solve this issue have included developing non-linear cantilevers and spring designs based on duffing resonators [7,8,9,10], impact driven mechanical stoppers [11,12,13], additional magnetic forces [14,15,16], bistable non-linear devices [17,18,19], and the designing of an array of devices with varying frequencies [4]. However, these methods have numerous disadvantages, such as hysteresis effects, which depend on frequency sweep testing protocol, low power density, as decreasing the Q-factor reduces the peak power harvested, a larger footprint, thus decreasing the overall power density, complex manufacturing, especially at the micro-scale, and the need for external power, which reduces the overall efficiency of the system. Impact-based systems causing non-linear dynamics were also recently investigated and have demonstrated an increase in bandwidth [20,21], but the methods investigated to date consist of impact methods that are not scalable down to the micro-scale needed for IoT applications.

Recently, there were numerous attempts to increase the bandwidth by using a lateral sliding mass, which changes the center of gravity of the proof mass during oscillation, resulting in a continuously changing resonant frequency in a process referred to as dynamic tuning [22]. This dynamic tuning has the effect of widening the bandwidth. Previous attempts to create a sliding mass include using rolling cylinders [23,24,25,26], sloshing liquids [22,27,28,29,30], and a combination of the two [31]. These wide bandwidth methods involve altering the resonant frequency during oscillation by changing the effective location of the proof mass. The various methods demonstrated limited success as they rely on a large change in the center of gravity to significantly increase the bandwidth. The large lateral displacements required to widen the bandwidth are potentially feasible in macro-scale devices, but scaling down to the micro-scale would limit lateral displacement of the movable mass. In addition, these devices increase the bandwidth when compared to a traditional linear (stationary proof mass) system, but altering the center of gravity has only a minor effect on the overall effective mass, which limits the bandwidth enhancement capabilities.

This paper investigated the experimental validation of a novel movable mass energy harvesting system aimed at widening the bandwidth. Instead of a lateral moving mass, this paper investigated a vertical or transverse movable mass component. The transverse movable mass concept combined: (i) a continuous alteration of the effective proof mass to change the resonant frequency during operation, and (ii) the non-linear dynamics based on the impact of the movable mass on the cantilever substrate, in order to increase the bandwidth without a significant decrease in power. This is the first time a vertical movable mass was embedded into the proof mass of an energy harvesting system. The development of a transverse movable mass has the potential to significantly alter the effective mass of the system, resulting in the continuous altering of the resonant frequency during operation. The transverse movable mass is potentially a preferred option over a lateral sliding mass because it provides a larger change in the overall mass of the system, which correlates to an increase in bandwidth. This method also has the potential to be applied to both macro- and micro-scale devices, as embedded powders in silicon cantilevers were previously developed [32], which could act as a movable mass for MEMS cantilevers. This paper uses a piezoelectric cantilever as the energy harvesting system, but the method of widening the bandwidth can be applied to any cantilever device. This paper investigated the power and bandwidth effects of a transverse movable mass cantilever-system by performing experimental testing associated with varying the acceleration, the amount of movable mass, the size of the movable mass, and varying the material density of the movable mass. The movable mass method under investigation was expected to be utilized for applications requiring large acceleration and low frequency, such as aerospace, automotive, or pacemaker applications [33,34]

## 2. Materials and Methods

### 2.1. Concept

The simplistic equation for determining the resonant frequency of a rectangular cantilever with stationary proof mass is given by the equation [35]:

(1) $$f=\left(\frac{1}{2\pi }\sqrt{\frac{E}{4m}}\right)\sqrt{\frac{{\mathrm{wt}}^{3}}{L^{3}}}$$

where E is the elastic modulus of the beam, m is the mass, and w, t, and L are the width, thickness, and length of the beam, respectively. The resonant frequency can be altered by changing the physical dimensions of the cantilever, the elastic modulus, or the mass. Changing the elastic modulus of the beam was previously attempted as a tuning method [36], but it is difficult to implement in practical applications. Changing the dimensions of the beam during operation is not feasible. Therefore, changing the effective mass to alter the resonant frequency is the most feasible option. A vertical moving mass will not change the center of gravity, but instead works by altering the overall effective mass during operation as the movable mass can be in free fall, which effectively eliminates its contribution to the overall mass at that specific time. Therefore, the cantilever will effectively have two resonant frequencies: (1) when the movable mass is on the surface contributing to the effective mass, and (2) when the movable mass is in free fall, this will result in two separate resonant frequencies. If the weight percent (wt.%) of the movable mass is large, then the two frequencies will have a wide range. If the system has multiple movable masses, then it will have multiple resonant frequencies, thus resulting in a wide bandwidth effect. Numerically, the change in resonant frequency from the movable mass is given by the following equation:

(2) $$\frac{f^{\prime }}{f}=\frac{\left(\frac{1}{2\pi }\sqrt{\frac{E}{4m^{\prime }}}\right)\sqrt{\frac{{\mathrm{wt}}^{3}}{L^{3}}}}{\left(\frac{1}{2\pi }\sqrt{\frac{E}{4m}}\right)\sqrt{\frac{{\mathrm{wt}}^{3}}{L^{3}}}}$$

where m’ is the mass of the stationary proof mass and m is total mass of the stationary proof mass plus the movable mass. If all other properties except the mass remain constant, the normalized increase in resonant frequency, by altering the effective proof mass while the movable mass is in free fall, can be simplified to:

(3) $$\frac{f^{\prime }}{f}=\frac{\sqrt{\frac{1}{m^{\prime }}}}{\sqrt{\frac{1}{m}}}$$

where f’ represents the frequency when the movable mass is in free fall and f represents the frequency when both masses are stationary. Thus, a 50% reduction in mass during free fall would result in, approximately, a 1.4× increase in the resonant frequency. Therefore, having higher wt.% of movable mass will further increase the frequency range, resulting in a wider bandwidth. Having multiple moving masses would then create multiple resonant frequencies, which would effectively increase the bandwidth, and more movable mass would generate an increased change in resonant frequency, thus resulting in a larger bandwidth. However, for the movable mass to contribute to the change in frequency, the acceleration force needs to be large enough to propel the movable mass from the surface.

**Figure 1:**
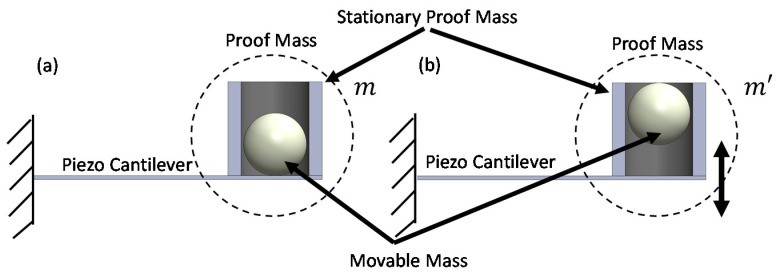
Schematic of the concept for transverse vertical movable mass demonstrating the various modes of operation: (**a**) demonstrates when the cantilever is at rest and the overall proof mass consists of the stationary proof mass and the movable mass, and (**b**) demonstrates when the cantilever has a large enough displacement to propel the movable mass off the substrate so the effective proof mass is represented only by the stationary proof mass.

The concept of creating a vertical movable mass was briefly described in our conference paper [37]. The experimental setup for validating the concept included a stationary proof mass with single or multiple chamber(s)/cavities that could be partially filled with a solid movable mass. The chamber was designed to promote vertical movement and prevent lateral movement of the solid mass. As the cantilever beam oscillates with a large tip displacement, the movable mass will be propelled off the cantilever substrate and thus be in free fall. While the movable mass is in free fall, the effective mass of the system is significantly reduced depending on the wt.% of the movable mass. Therefore, during operation, the cantilever is continuously altering its resonant frequency and, thus, effectively widening the bandwidth. Figure 1 demonstrates the concept by having a solid movable sphere mass within a stationary proof mass. While at rest (Figure 1a) the overall mass of the system consists of: (i) a stationary proof mass chamber, and (ii) the mass of the solid sphere. The mass of the cantilever is considered negligible. The second mode of operation is when the cantilever displacement is large, causing the movable mass to displace as shown in Figure 1b. In this mode the effective mass only consists of the stationary mass, as the solid movable mass is in free fall. This concept also involves an impact mode (not shown in Figure 1), which creates the non-linear dynamic effect. The impact is caused from the solid movable mass falling, due to gravity, and impacting the cantilever substrate. The effect of the impact on the resonant frequency will depend on the force of the impact, which is determined by the vertical displacement of the movable mass, the density of the mass, and the stiffness of the substrate. This concept was designed for low frequency, high acceleration applications, as the concept requires a large displacement of the cantilever to generate enough force to move the solid mass. The energy harvesting operation uses a piezoelectric cantilever that generates stress and strain during oscillation, which then converts mechanical energy into electrical energy.

The system involves non-linear dynamics due to both the impact as well as the continuous change in the effective mass of the system. In addition, if multiple movable masses are in a chamber, then there is an influence from the impact forces between the movable masses as well as when they impact the substrate. When the movable mass is stationary or at rest, the effective mass of the system will be significantly higher than when the movable mass is in free fall (depending on the density and wt.% of the movable mass). With the addition of the movable mass component, the resonant frequency of the system at rest will be significantly lower than the resonant frequency while the movable mass is in free fall (dependent on the wt.% of movable mass). Thus, the system will begin to oscillate at a lower frequency, which will cause the movable mass to move out of plane. Since the time for the movable mass to fall and the time for the cantilever to complete a cycle are different, the system will be going in and out of resonant frequency, and additional frequencies generated from the impact of mass will cause the system to be non-linear. Modelling the complete dynamics of the entire system are beyond the focus of this paper, which focuses instead on the experimental validation of the concept.

### 2.2. Cantilever Structure

**Figure 2:**
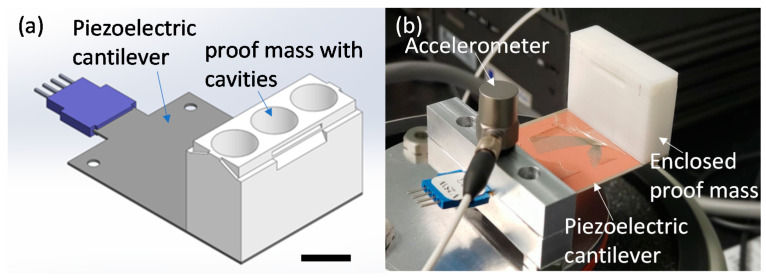
Image of the cantilever system: (**a**) schematic of the piezoelectric cantilever and custom designed proof mass with three chambers for the movable mass (the top lid is not shown), and (**b**) image of the cantilever with proof mass and cap with accelerometer mounted on vibration shaker. Scale bar is 1 cm.

In this paper, a commercial piezoelectric energy harvester (Volture V25W, Mide), with dimensions of 4.6 × 3.8 × 0.06 cm (l, w, and t), was used as the cantilever structure to validate the concept at the macro-scale. The stationary proof mass, which was 1.5 × 3.8 × 2.5 cm (l, w, and t) in size, was custom designed and 3D printed (Ultimaker S5) using PLA. The lightweight PLA was used to reduce the mass of the stationary proof mass to ensure that the wt.% of the movable mass was high. The dimensions of the proof mass and cantilever are scaled up versions of typical microsystem energy harvester devices [1,15,38]. The 3D printed proof mass consisted of three 11 mm diameter hollow chambers along the width of the cantilever, designed to restrict the 10 mm diameter spheres from rolling in the lateral direction. These chambers had a height of 20 mm and a base thickness of 5 mm to allow the movable mass to impact the proof mass instead of directly impacting the cantilever, which could lead to mechanical failure. The chambers were symmetrically aligned on the cantilever. A lid was 3D printed to prevent the movable mass from exiting the chamber. The overall mass of the 3D printed stationary proof mass was ~10 g. Figure 2 is a schematic and image of the cantilever with the custom proof mass. The proof mass was attached to the cantilever using double-sided adhesive tape.

The movable mass in our investigation consisted of solid spheres that were 10 mm in diameter, 5 mm diameter balls were also investigated to determine the effect of varying ball dimensions. The balls, which had a density of 14.95 g cm ^−3^, were made from tungsten carbide (labelled W in the paper; MSE Supplies), the main type of material investigated because of its large density since, theoretically, the bandwidth should be dependent on the wt.% of the movable mass. According to Equation (3), the higher percentage of movable mass should generate a wider range of resonant frequencies between the stationary resonant frequency and the free fall resonant frequency, which should correlate to a larger increase in bandwidth. Other materials, including stainless steel 316L (SS, 8 g cm^−3^; GoodFellow), Aluminum oxide (Al_2_O_3,_ 3.9 g cm^−3^), and Teflon (PTFE, 2.2 g cm^−3^; United States Plastic), were investigated to determine the effects of changing the wt.% of the movable mass by changing the density of the balls. The outer surfaces of the balls were polished to reduce friction between the balls and the surface of the proof mass.

### 2.3. Experimental Methodology

**Figure 3:**
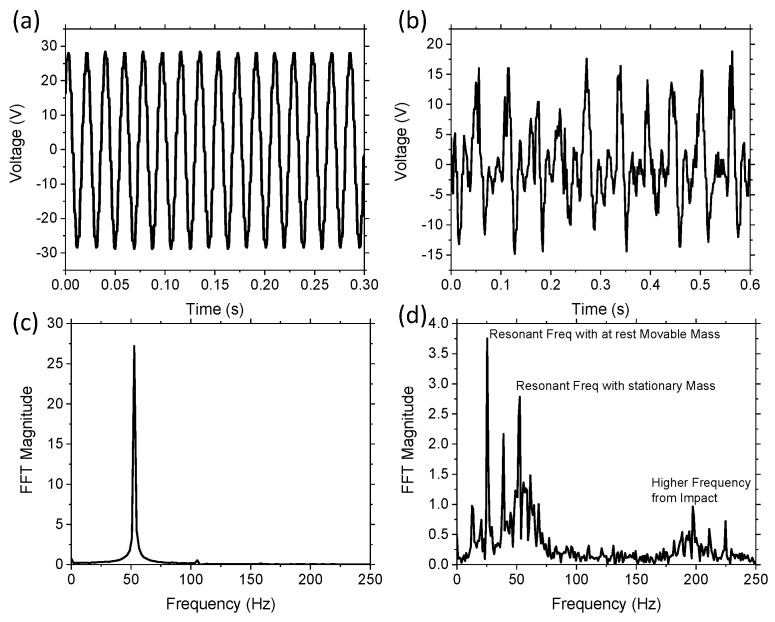
Experimental raw data at 1 g acceleration for (**a**) the control device with stationary proof mass and no movable mass, and (**b**) a device with one movable W ball and an off resonance applied frequency; (**c**,**d**) are the FFT of the raw data, where (**c**) is the FFT of the linear control device, and (**d**) is the FFT of a non-linear device with multiple frequencies.

The cantilever device with proof mass was mounted on a vibration shaker (ET 126, Labworks) via a custom-made mounting chuck with an accelerometer to provide acceleration feedback control. The system from Labworks has an integrated accelerometer and vibration control software. The cantilever was connected to an oscilloscope (Tektronix 4-channel 100 MHz with 5 GS/s sampling rate) with a variable load resistor to provide impedance matching for power measurements. Power was calculated based on the V_rms_ and impedance. The optimal power was determined by matching the electrical impedance with the impedance of the piezoelectric cantilever. Discrete manual frequency sweeps were performed in 0.5 Hz steps. A sweep up and down test was performed to determine if sweeping had any impact. In addition, random frequency values were tested and compared to the sweep data to determine if sweeping had any impact on the power and bandwidth. Once the force of the cantilever was large enough to cause vertical displacement of the balls within the chambers, the oscilloscope readings became non-linear due to the impact force, meaning the voltage and power amplitude varied with time and were no longer a simple sinusoidal waveform. The average V_rms_ value, over the course of 1 min at a specific frequency, was measured during the non-linear dynamic range. The frequency range of the impact from the movable mass is called non-linear bandwidth (NLBW), as it is essentially bandwidth at which the movable mass operates, resulting in non-linear dynamics from the impact. The start and end frequency of the movable mass, which is the frequency range at which the impact from the movable mass was observed quantitatively through data from the oscilloscope, as voltage output was no longer sinusoidal, but instead consisted of multiple frequency outputs as demonstrated in Figure 3b. The frequency range of the movable mass could also be determined through hearing the impact of the ball. The NLBW was essentially the frequency range at which the movable mass had significant displacement, causing the mass to be propelled off the surface and impact the substrate.

Experimental testing used W 10 mm diameter balls, unless specified, while 5 mm diameter balls were used to determine the effects of different movable mass dimensions. An investigation of the effect of the number of balls used, or the total amount of movable mass, consisted of comparing power vs. frequency measurements for a device with three balls (one in each chamber) and a device with a single ball in the middle chamber. The effects of acceleration on the bandwidth and power magnitude were determined using accelerations of 0.1, 0.5, 1, and 1.5 g. To determine the effects of changing the wt.% of movable mass, different materials were investigated with varying accelerations as shown in Table 1. The wt.% of the movable mass was calculated as the amount of movable mass divided by the total mass, which included the mass of the balls and the stationary proof mass. The amount of movable mass varied from 22.5% to 70.2%. To determine the effect of the size of the balls, we compared systems with one 10 mm W sphere vs. eight 5 mm W spheres, as they had nearly the same movable mass component of 7.87 g and 7.84 g, with a moveable mass percentage of 44% and 43.9%, respectively, as shown in Table 1, which was considered equivalent.

## 3. Results and Discussion

The initial validation of the concept was investigated experimentally by comparing the output voltage as a function of time for two devices operating at 1 g acceleration. The first device was the control, which consisted of the cantilever beam and a stationary 3D printed proof mass without any movable balls in the chambers. The second device consisted of a cantilever beam, stationary proof mass, and one W ball in the middle chamber. In both cases, the vibration source was applied at the resonant frequency of the cantilever with proof mass. The control proof-mass was lighter, having a resonant frequency of ~53 Hz, whereas, when we added the extra mass of the movable ball, the resonant frequency was ~25 Hz.

Figure 3a is a section of the raw data from the oscilloscope while operating near the resonant frequency of the control device with no movable component. The movable mass was restricted from moving in the lateral direction due to the size of the chamber, and thus allowed the ball to only move in the vertical transverse direction. The results of the control system with no movable mass demonstrated a typical linear device with a sinusoidal output. The FFT of these results demonstrated a single resonant frequency at 53 Hz, as shown in Figure 3c. The experimental raw data for the device with movable mass is shown in Figure 3b. The results demonstrate that the movable mass generated an output voltage consisting of multiple frequencies, with a peak resonant frequency of approximately 25 Hz. The reduction in resonant frequency was due to the increased overall proof mass. The large voltage peaks consisted of linear frequencies when the movable mass was in contact with the cantilever and when the system was in free fall. Visual confirmation of the ball moving was seen through the chamber and the ball displacement was approximately 1 cm in height. The FFT of the movable mass device is demonstrated in Figure 3d, which shows multiple frequencies, including a peak at 53 Hz. The 53 Hz peak is demonstrated in both Figure 3c,d, as this represents the resonant frequency of the cantilever with the stationary 3D printed mass. Figure 3d also has a large peak at 25 Hz, which is the resonant frequency of the cantilever with the additional W mass while at rest on the surface. Numerical analysis using Equation (1) estimates the resonant frequency of the two masses to be 26 Hz and the free fall state resonant frequency to be 52 Hz, which is in good agreement with the experimental results. The difference was likely to be a result of a slight difference in the elastic modulus of the cantilever. The FFT data demonstrated multiple frequencies in the 10–75 Hz range, even though the excitation frequency was at 25 Hz, which was due to the non-linear impact of balls and the changing resonant frequency of the cantilever. There were also higher frequency peaks at around 175–225 Hz, which was due to the impact of the ball hitting the chamber. The results validate that there was a wider range of frequencies occurring in the system with the movable mass. The addition of these multiple frequencies should have the effect of increasing the bandwidth, as significant voltage output can be measured through a wide range of frequencies compared to the linear control system in Figure 3c, which only has a single frequency output.

**Figure 4:**
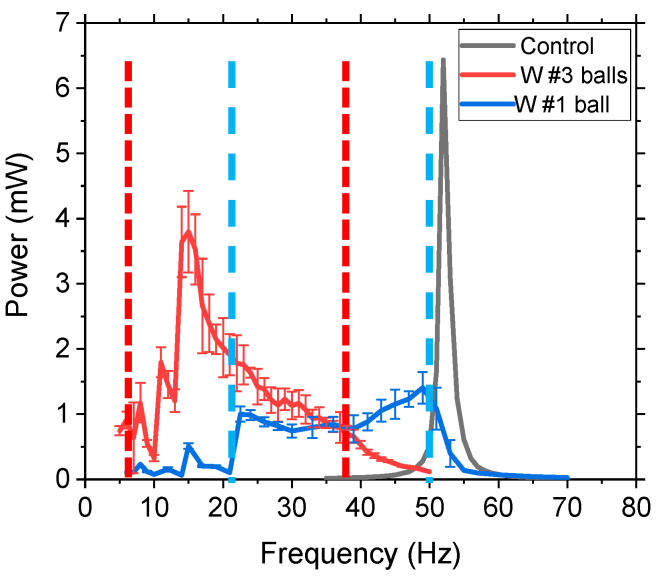
Experimental data of power as a function of frequency for 10 mm W balls at 1 g acceleration for systems with zero (control), one, and three movable balls. The error bars represent the standard deviation of the power measurements during the 1 min testing duration. The dashed lines represent the start and stop frequencies for the movable mass representing the NLBW.

Next, we varied the number of balls in each chamber to determine if the wt.% of movable mass had a significant impact on bandwidth. The power, as a function of applied frequency, was measured for the (i) control (no movable mass), (ii) one middle 10 mm diameter W ball, and (iii) three W balls (one in each chamber), which were experimentally investigated at 1 g acceleration. The power was measured by connecting the output voltage of the cantilever to a variable load resistor to match the impedance for maximum power. The results are demonstrated in Figure 4. The control system had a typical linear dynamic response, with a sharp peak and full width half maximum (FWHM) of 1.5 Hz, and a peak power of 6.43 mW at 53 Hz. The movable mass device (a single W ball), with a 44% movable mass, had a wider bandwidth with a peak frequency shifted to the left (lower frequency) due to the additional mass in the system. The average power was calculated for each frequency over a 1 min duration, and the standard deviation was represented by error bars. The single ball system had a much wider bandwidth compared to the control, which had no movable mass component, and a FWHM (~30.5 Hz compared to 1.5 Hz) and no distinguishable peak, but the average power measured was 1.4 mW. The results of adding a single ball with a 44 wt.% movable mass significantly increased the bandwidth, but decreased the power. The three-ball system, with a 70.2 wt.% movable mass, demonstrated a high bandwidth and a high peak power of 3.8 mW at 15 Hz. The area under the curve, which represents the power over a range of frequencies (mW-Hz), was increased by 117% and 252% for the one-ball and three-ball system, respectively, compared to the control. The three-ball system had a NLBW range of 34 Hz, which demonstrated average power values of >0.7 mW during that frequency range, with the exception of one point around 10 Hz. This validated the concept that a vertical movable mass can widen the bandwidth and increase power generation. The peak power of the movable mass system was reduced compared to the control, but the bandwidth was significantly increased. Increasing the percent of the movable mass further increased the peak power, while still maintaining a high bandwidth as the power over a frequency range was significantly increased. Balls placed on the outside chambers caused high frequency twisting modes to decrease their resonant frequency as well, but the research was interested in low frequency first mode operation to increase the power density.

**Figure 5:**
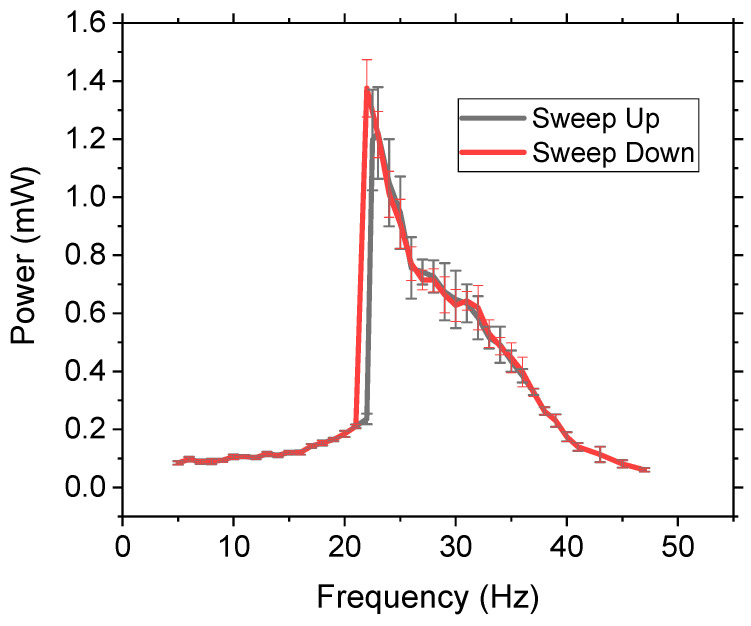
Comparison of sweep up and sweep down frequency testing on the system with three W balls of 10 mm at 0.5 g. Error bars are equal to one standard deviation.

The non-linear energy harvester’s bandwidth and power measurements are commonly dependent on the frequency sweep characteristics of the applied excitation where there is a hysteresis effect; thus the operation of the device is dependent on the testing parameters of sweeping the frequency either up or down [39,40,41]. However, this is not practical as most applications have randomly changing discreet frequencies instead of a continuous sweep. To investigate if the frequency sweep direction had an impact on the power or bandwidth, the frequency was swept in both directions at a rate of 0.05 Hz/s from 5–50 Hz at 0.5 g with a three-ball system of W. The results (Figure 5) demonstrated no significant difference in amplitude or bandwidth due to sweeping the frequency. The results were further validated by randomly selecting frequencies and measuring their output power, which demonstrated that, within the standard deviation, the results were the same. Therefore, this method has potential impact in real-life applications as the power and bandwidth can be accurately predicted regardless of testing parameters. Comparing the results of the three-ball system in Figure 4 (1 g) and Figure 5 (0.5 g) the general shape of the output power vs. frequency was the same, but the 1 g acceleration had higher power, as expected, and higher bandwidth due to the increased impact force. Increasing the acceleration to 1 g and 1.5 g obtained similar results, demonstrating no significant hysteresis effect.

**Figure 6:**
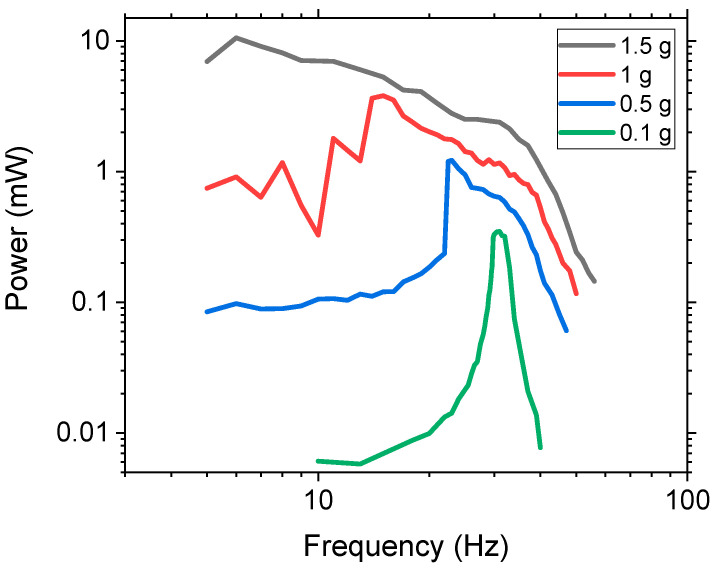
Log scale power as a function of frequency for varying acceleration with three W balls.

The power and bandwidth are dependent on the acceleration that is applied, and it was believed that the vertical movable mass method required high acceleration because low acceleration would not have a large enough tip displacement to propel the mass within the chamber, thereby limiting the non-linear dynamic effects. To investigate this, various accelerations from 0.1, 0.5, 1, and 1.5 g were experimentally tested with the three-ball system using W balls. The results are presented in Figure 6. At 0.1 g, the system behaved like a linear system with a slight increase in the FWHM of 1.73 Hz as compared to the control’s 1.2 Hz. As the acceleration increased, there was a shift in the peak power amplitude towards a lower frequency. This occurred because the higher acceleration resulted in higher force, which increased the tip displacement and caused the movable mass to propel off the surface at lower frequencies. This, in turn, increased the NLBW as the frequency range of the movable mass was increased, thus generating a larger non-linear region due to the impact force. Once the mass started moving, the peak power increased along with the bandwidth. At 1.5 g, the peak power occurred at 6 Hz because the high acceleration and low frequency produced a large enough force to propel the movable mass off of the substrate. This resulted in a peak power of 10.52 mW. Higher acceleration also caused the bandwidth to increase as the area under the curve increased from 1.73, 13.99, 53.91, and 147.23 mW-Hz for 0.1, 0.5, 1, and 1.5 g, respectively. The area under curve represents the total amount of power that is harvested over a range of frequencies. The experimental results demonstrated that this method is efficient for low frequency, high acceleration applications.

**Figure 7:**
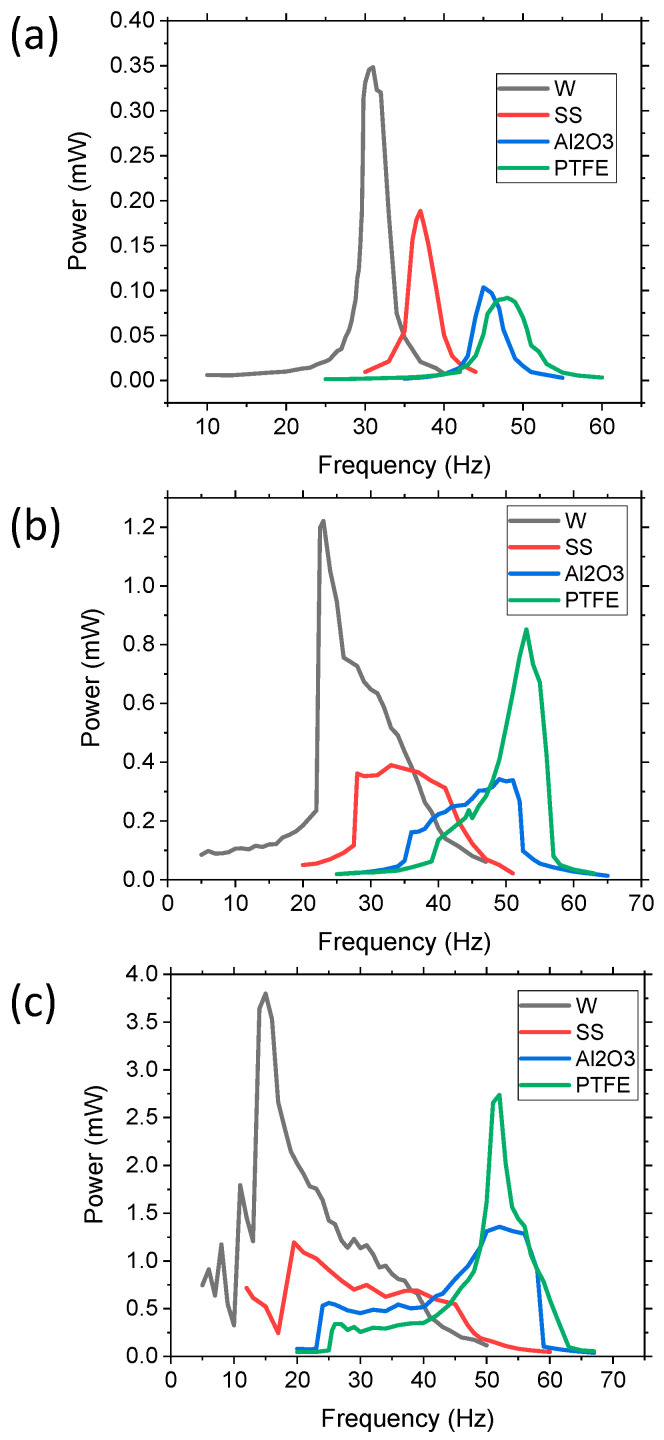
Experimental results of varying ball materials (densities) with varying accelerations: (**a**) 0.1 g, (**b**) 0.5 g, and (**c**) 1 g. Each test consisted of three balls of 10 mm diameter of W (tungsten), SS (stainless steel 316L), Al_2_O_3_ (aluminum oxide), or PTFE (Teflon).

Various density balls, as highlighted in Table 1, were used to determine the effects varying the wt.% of movable mass had on the bandwidth and power. Three 10 mm diameter balls, one in each chamber, were used in the experiment with varying accelerations from 0.1, 0.5, and 1 g. The results are shown in Figure 7. At a low acceleration of 0.1 g (Figure 7a), all of the materials used for the balls (W, SS, Al_2_O_3_, and PTFE) had an output that more closely represented a linear system. As the density of the balls increased, there was a peak power shift towards lower frequencies, which was expected since the overall weight of the proof mass increased. The peak power was dependent on the density of the balls and the overall mass of the system, as expected, demonstrating an increase in the power with an increase in the percentage of movable mass. However, the FWHM value decreased as the amount of movable mass increased, with values of 2.61, 3.01, 3.7, and 5.2 Hz for W, SS, Al_2_O_3_, and PTFE, respectively, compared to the control’s FWHM of 1.2 Hz. The PTFE balls had a wider bandwidth at a low acceleration because they required less force to propel as compared to the denser masses, such as W, which required more force. Therefore, low-density movable masses provided wider bandwidths than dense materials at low acceleration because the higher density balls did not easily propel off the substrate, resulting in limited impact force.

As the acceleration increased, the cantilevers with denser masses demonstrated an increase in bandwidth and all of the systems had a larger NLBW, as shown in Figure 7b,c, because the acceleration produced a large enough force to propel the movable mass. For example, at 0.5 g, the W balls had a high intensity peak (1.22 mW) at a low frequency and a FWHM of 9.29 Hz. However, it started to develop a broader peak at higher frequencies, thus increasing the bandwidth of usable energy. Usable energy, as defined by the authors, is power >0.7 mW, which was determined based on the typical power requirements for wireless sensor networks, wherein 0.7 mW is adequate for most IoT applications. However, at 0.5 g, the SS balls lacked a high-intensity, narrow peak, instead consisting of a broad peak with an average power of 0.38 mW and a bandwidth of 15.4 Hz. The Al_2_O_3_, on the other hand, had a broad peak at lower frequencies and started to develop a high-intensity peak at the resonant frequency of the stationary system. This resulted in an average power of 0.25 mW and a bandwidth of 14.52 Hz. The PTFE movable mass system had a high-intensity peak at the resonant frequency of the stationary proof mass, with a peak power of 0.85 mW and a FWHM of 6.81 Hz, but it also consisted of a wider peak at lower frequencies due to the non-linear movable masses. The reason for the high peak exhibited by PTFE was attributed to two issues: (1) the low density of the balls caused a lower impact as compared to W, so the non-linear effects were less, and (2) the less-dense balls had a larger displacement within the chamber, thus the time required for the balls to impact the substrate was longer, resulting in a larger linear dynamic region for a longer amount of time. For example, if the PTFE balls had a 1.5 cm vertical displacement within the chamber the free fall, the time it would take for the balls to fall would be ~55 ms, whereas the time for the cantilever to complete one cycle is ~19 ms, thus the cantilever would be in linear mode for nearly three oscillation cycles before the impact. The W ball system had a large narrow peak at low acceleration due to the cantilever’s limited amount of force and displacement, which resulted in the low displacement of the balls and a low impact force. As acceleration increased, the balls had a larger displacement, resulting in a larger NLBW region. The denser balls had a larger impact force when they were propelled. Therefore, the low-density movable masses resulted in a more linear system, but at high acceleration, the system had both linear and nonlinear components.

The power, as a function of the frequency results, of applying a 1 g acceleration (Figure 7c) demonstrated a similar trend as the results in Figure 7b. The Al_2_O_3_ ball appeared to be shifting in the same manner as the PTFE samples to form a peak at the linear resonant frequency. At 1 g, however, a significant narrow peak was still absent, but increasing the acceleration is likely to cause a rise in the peak at the stationary resonant frequency. Both PTFE and Al_2_O_3_ had two distinguished modes: (i) a wide plateau mode at lower frequencies of 20–45 Hz approximately (non-linear region), and (ii) a higher intensity peak region (linear). This resulted in the two systems having both a narrow high-power density section (the linear component) and a lower power density wider bandwidth section (the non-linear component). The total power over the usable frequency range resulted in an area under curve value of 28.07 and 28.8 mW-Hz for Al_2_O_3_ and PTFE, respectively. SS and W also increased the bandwidth and power, with the SS balls having an average power of 0.7 MW and a FWHM of 29.9 Hz. The W balls had a large peak power of 3.79 mW and a wider NLBW region, with an overall area under the curve of 54.07 mW-Hz. Therefore, we determined that a higher wt.% movable mass provided a wider bandwidth and higher power at increased acceleration. Applying a higher acceleration to the W ball system would likely cause the output to shift, similar to the outputs of PTFE and Al_2_O_3_, but we maintained the acceleration limit at 1 g as most practical applications operate at <1 g acceleration.

**Figure 8:**
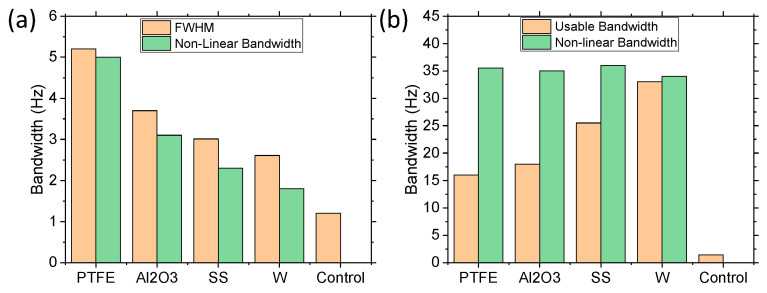
Bandwidth comparison of varying ball materials at (**a**) 0.1 g and (**b**) 1 g, demonstrating the FWHM bandwidth and the NLBW (range of frequency of movable masses).

Figure 8 demonstrates the bandwidth effects of the varying materials from 0.1 g to 1 g. The results of the 0.1 g bandwidth investigation (Figure 8a) were measured by the FWHM (as these devices behaved more like a linear system) and the NLBW, as the devices still had some movable mass components. For low acceleration, the FWHM values were similar to the NLBW range, with the NLBW being slightly reduced in all cases. However, the difference between the FWHM and NLBW increased as the density of the balls increased, which made sense since the denser balls had a larger impact force on the cantilever. In all cases, the bandwidths were significantly larger than the control with no movable mass, thus validating the concept. The bandwidth also increased as the density of the balls or total movable mass decreased because the lower density balls had larger displacements at the lower acceleration force, whereas the low acceleration force was unable to propel the higher density balls from the surface. However, as the acceleration was increased to 1 g (Figure 8b), the NLBW approached a steady state, with all materials having similar bandwidth of 34–36 Hz. The FWHM values did not apply to the non-linear systems as they have large peaks and wide band regions, thus the term usable bandwidth was employed to represent bandwidths with a power of >0.7 mW. The usable bandwidth increased as the amount of movable mass increased. As the 1.5 g W balls demonstrated an NLBW of 43 Hz, therefore demonstrating that the saturated NLBW was dependent on the acceleration applied, it was determined that the NLBW could be further increased by increasing the acceleration.

**Figure 9:**
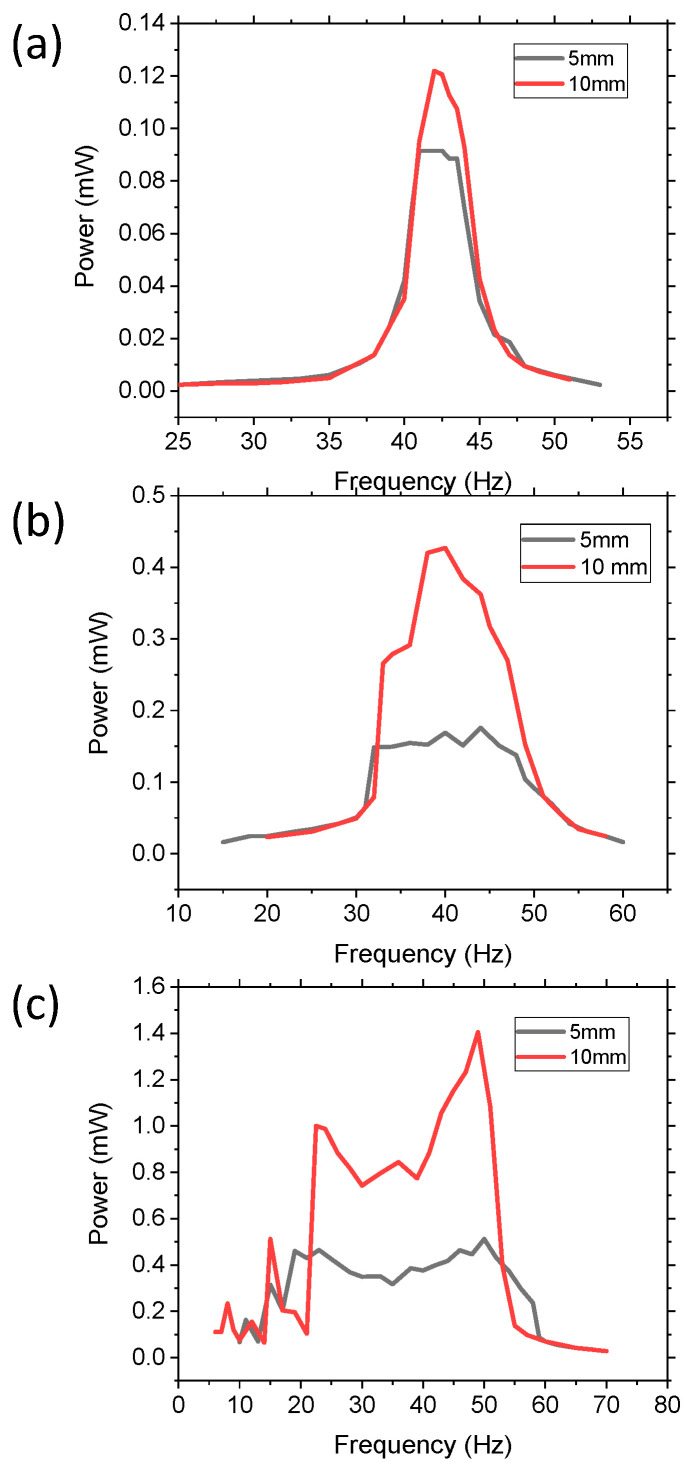
Demonstrating the effect of varying the size of the movable mass, while keeping movable mass percentage the same, by using one 10 mm W ball and eight 5 mm W balls and varying the acceleration from (**a**) 0.1 g, (**b**) 0.5 g, and (**c**) 1 g.

The effect of the physical dimensions of the movable masses on power and bandwidth were investigated by comparing devices with (i) one 10 mm diameter W sphere with mass of 7.87 g and (ii) eight 5 mm W spheres with mass of 7.84 g. The density of the balls was the same and only the diameter changed (5 mm vs. 10 mm). By increasing the number of 5 mm diameter balls, the two devices had approximately the same wt.% of movable mass, differing only by 0.1%. The effects with varying acceleration from 0.1, 0.5, and 1 g are demonstrated in Figure 9. In all three accelerations, the larger diameter movable mass had higher power harvesting capabilities, but the bandwidth of the two devices was the same for an applied acceleration. For 0.1 g, the devices acted like a linear system with peaks of 0.122 and 0.09 mW and a FWHM of 3.56 and 3.85 Hz, compared to the control’s 1.2 Hz FWHM. At 0.5 g, the devices had more non-linear dynamic properties and had peak power values of 0.43 mW and 0.17 mW, with a bandwidth of 16.24 and 17.3 Hz for the 10 mm and 5 mm balls, respectively. At 1 g, the peak power increased to 1.40 mW and 0.51 mW and the bandwidth increased to 38.1 Hz and 41.2 Hz. For each acceleration, there was a slight increase in bandwidth for the smaller balls, but it was not statistically different. However, the power difference between the various accelerations was significant. Therefore, the size of the movable mass did significantly impact the amount of power harvested, but it did not significantly affect the bandwidth. The bandwidth was, thus, dependent on the percent of movable mass as well as the applied acceleration.

## 4. Conclusions

In conclusion, we successfully validated a novel concept of widening the bandwidth of a cantilever device by creating an embedded vertical or transverse movable mass system. The results demonstrated that the bandwidth and power effects were dependent on the acceleration and the amount, size, and density of the movable mass. The size of the movable mass directly affected the amount of power that could be harvested, but it did not have a significant effect on bandwidth. However, the density of the movable mass, which affected the overall wt.%, significantly affected both the power and bandwidth, with lower density masses preferred for low acceleration applications.

In the end, all of the abovementioned parameters influenced the bandwidth and amount of power, and further investigation is required to optimize the parameters for a specific application. By modifying these parameters, the end user may fine tune the bandwidth and power specifications to match the specifications of the application. The concept of a vertical movable mass system demonstrated a significant increase in bandwidth for high acceleration, low frequency applications. However, the concept can be implemented in low acceleration applications by using less dense movable masses. The power magnitude was dependent on the size, density, and overall percentage of the movable mass component. The dynamics of the system consisted of both linear and non-linear components, which requires further investigation in the future to enhance device performance. The vertical movable mass method described in this paper has potential use in both macro- and micro-scale cantilevers, but the concept requires further investigation at the micro-scale level to demonstrate validity.

This novel method provides researchers with an alternative means of widening the bandwidth without significantly decreasing power, and it has potential to be scaled down to the micro-scale to power IoT applications. The concept was experimentally validated using a piezoelectric energy harvesting cantilever, but the concept could also be applied to other energy harvesting systems, such as electromagnetics and electrostatics as well as other cantilever applications that require a wide bandwidth. This method is unique as it provides increased bandwidth, while also providing high peak power, demonstrating it can potentially provide both high power and high bandwidth. However, the results are dependent on various parameters and would need to be optimized to the specific application.

## 5. Patents

An invention titled “Enhanced Frequency Bandwidth of Cantilever Beam using Transverse Movable Mass,” was filed through the USPTO.

## Figures and Tables

**Table 1:** Various scenarios of movable mass and amount of movable mass used in each test.

Material	Mass of One Ball (Grams)	Quantity of Balls	Movable Mass (Grams)	Movable Mass (%)
Tungsten Carbide (W)	7.87	3	23.61	70.2
Tungsten Carbide (W)	7.87	1	7.87	44.0
Tungsten Carbide 5 mm ball	0.98	8	7.84	43.9
SS 316L (SS)	4.168	3	12.50	55.6
Al_2_O_3_	1.76	3	5.28	34.6
PTFE	0.97	3	2.91	22.5
PTFE	0.97	6	5.82	36.8

## Data Availability

Data is contained within the article.
